# Dissemination of IncF-type plasmids in multiresistant CTX-M-15-producing Enterobacteriaceae isolates from surgical-site infections in Bangui, Central African Republic

**DOI:** 10.1186/s12866-015-0348-1

**Published:** 2015-02-04

**Authors:** Clotaire Rafaï, Thierry Frank, Alexandre Manirakiza, Alfred Gaudeuille, Jean-Robert Mbecko, Luc Nghario, Eugene Serdouma, Bertrand Tekpa, Benoit Garin, Sebastien Breurec

**Affiliations:** Institut Pasteur, Laboratory of Bacteriology, Avenue de l’Independance, BP 923, Bangui, Central African Republic; Institut Pasteur, Unit of Epidemiology, Avenue de l’Independance, BP 923, Bangui, Central African Republic; Complexe Pédiatrique, Department of Paediatric Surgery, Avenue de l’Independance, Bangui, Central African Republic; Hôpital de l’Amitié, Department of General Surgery, Avenue de l’Independance, Bangui, Central African Republic; Hôpital de l’Amitié, Department of Gynaecology and Obstetrics, Avenue de l’Independance, Bangui, Central African Republic; Hôpital Communautaire, Department of Orthopaedic Surgery, Avenue des Martyrs, Bangui, Central African Republic; Institut Pasteur, Laboratory of Bacteriology, BP 1274, Antananarivo, Madagascar; University of Antilles, Faculty of Medecine, Pointe-à-Pitre, French West Indies

**Keywords:** Antimicrobial resistance, CTX-M-15, IncF, Surgical-site infection, Enterobacteriaceae, Africa

## Abstract

**Background:**

Surgical-site infection is the most frequent health care-associated infection in the developing world, with a strikingly higher prevalence than in developed countries We studied the prevalence of resistance to antibiotics in Enterobacteriaceae isolates from surgical-site infections collected in three major tertiary care centres in Bangui, Central African Republic. We also studied the genetic basis for antibiotic resistance and the genetic background of third-generation cephalosporin-resistant (3GC-R) Enterobacteriaceae.

**Results:**

Between April 2011 and April 2012, 195 patients with nosocomial surgical-site infections were consecutively recruited into the study at five surgical departments in three major tertiary care centres. Of the 165 bacterial isolates collected, most were Enterobacteriaceae (102/165, 61.8%). Of these, 65/102 (63.7%) were 3GC-R, which were characterized for resistance gene determinants and genetic background. The *bla*_CTX-M-15_ and *aac(6′)-Ib-cr* genes were detected in all strains, usually associated with *qnr* genes (98.5%). *Escherichia coli,* the most commonly recovered species (33/65, 50.8%)*,* occurred in six different sequence types, including the pandemic B2-O25b-ST131 group (12/33, 36.4%). Resistance transfer was studied in one representative strain of the resistance gene content in each repetitive extragenic palindromic and enterobacterial repetitive intergenic consensus sequence-PCR banding pattern. Plasmids were characterized by PCR-based replicon typing and sub-typing schemes. In most isolates (18/27, 66.7%), *bla*_CTX-M-15_ genes were found in incompatibility groups F/F31:A4:B1 and F/F36:A4:B1 conjugative plasmids. Horizontal transfer of both plasmids is probably an important mechanism for the spread of *bla*_CTX-M-15_ among Enterobacteriaceae species and hospitals. The presence of sets of antibiotic resistance genes in these two plasmids indicates their capacity for gene rearrangement and their evolution into new variants.

**Conclusions:**

Diverse modes are involved in transmission of resistance, plasmid dissemination probably playing a major role.

## Background

Third-generation cephalosporin-resistant (3GC-R) Enterobacteriaceae represent a major threat in both hospital and community settings worldwide [1]. The resistance is mediated mainly by acquired *extended-spectrum beta-lactamase* (*ESBL*) genes carried by mobile genetic elements such as plasmids and transposons. This situation is of great concern, as *ESBL* enzymes can hydrolyse almost all beta-lactams (except carbapenems and cephamycins) and are frequently associated with genes that confer resistance to several other classes of antibiotic. During the past decade, CTX-M enzymes have gradually replaced the classical TEM and SHV-type *ESBLs* in many countries [1]. Rapid international spread of CTX-M-15 has been associated with global dissemination of *Escherichia coli* clones, such as sequence type 131 (ST131) harbouring *bla*_CTX-M-15_ on incompatibility group (Inc) FII conjugative plasmids [2].

Plasmid-mediated quinolone resistance has emerged in Enterobacteriaceae, with three mechanisms described: Qnr, which mediates target protection; AAC(6′)-Ib-cr, which mediates drug modification; and OqxAB and QepA, which mediate drug efflux [3]. These mechanisms increase the minimum inhibitory concentration (MIC) of fluoroquinolones, thereby facilitating the selection of mutants with greater resistance in the presence of quinolones through sequential chromosomal mutations in genes coding for the target enzymes, DNA gyrase and DNA topoisomerase IV [3].

Surgical-site infection is the most frequent health care-associated infection in the developing world, with a strikingly higher prevalence than in developed countries [4]. The alarming global burden of avoidable complications resulting from unsafe surgery has been highlighted by the World Health Organization [5]. Few good-quality data are, however, available in Africa on the phenotypic antimicrobial resistance of strains associated with surgical-site infections, and the mechanisms underlying antimicrobial resistance are unknown. We studied the prevalence of resistance to antibiotics in Enterobacteriaceae isolates from surgical-site infections collected in three major tertiary care centres in Bangui, Central African Republic; we also studied the genetic basis for antibiotic resistance and the genetic background of 3GC-R Enterobacteriaceae.

## Results

### Patients, antibiotic prophylaxis, bacterial strains and antibiotic susceptibility

A total of 195 patients (97 males, 98 females; mean age, 30.9 years; median age, 29.0 years; interval, 1.0–85.0 years) with surgical-site infections were included during the study period in the general surgical departments of the Complexe Pédiatrique (*n* = 33), the orthopaedics department of the Community Hospital (*n* = 64), and the general surgery (*n* = 41), gynaecology (*n* = 46) and urology (*n* = 11) departments of the Amitié Hospital. Most of the operations were class I (110/195, 56.4%) in the Altemeier classification, mainly with a pre-operative physical status score of I (115/195, 59.0%). The duration of most operations did not exceed 2 h (98.0%). Antibiotic prophylaxis was administered to 193 patients (99.0%), most frequently consisting of beta-lactams (92.3%), mainly ceftriaxon (93/193, 48.2%), penicillin G (*n* = 68, 35.2%) and ampicillin (*n* = 43, 22.3%).

Of the 195 non-duplicate biological samples taken, 151 were culture positive (77.4%). As 14 cultured specimens had mixed growth, a total of 165 bacterial isolates were collected. The bacteria isolated were *E. coli* (*n* = 47), *Staphylococcus aureus* (*n* = 44), *Enterobacter cloacae* (*n* = 18), *Acinetobacter baumanii* (*n* = 11), *Klebsiella pneumoniae* (*n* = 19)*, Pseudomonas aeruginosa* (*n* = 8), *Proteus mirabilis* (*n* = 8), *Morganella morganii* (*n* = 3), *Salmonella* spp. (*n* = 2), *Enterobacter aerogenes* (*n* = 2)*, Enterobacter amnigenus* (*n* = 1), *Enterobacter sakazakii* (*n* = 1) and *K. oxytoca* (*n* = 1).

Neither *P. aeruginosa* nor A. *baumanii* was resistant to imipenem, whereas 9% (4/44) of *S. aureus* isolates were methicillin-resistant, and 63.7% (65/102) of Enterobacteriaceae (33 *E. coli*, 16 *Enterobacter cloacae*, 10 *K. pneumoniae*, 2 *Proteus mirabilis*, 1 *M. morganii*, 1 *Enterobacter amnigenus,* 1 *Enterobacter sakazakii* and 1 *K. oxytoca*) were resistant to 3GC-R antibiotics. C3G-R Enterobacteriaceae strains were resistant to all the beta-lactams tested, except for cefoxitin (24.6% resistance, only *Enterobacter cloacae* strains), and all were susceptible to ertapenem (MIC, < 0.008–0.25 mg/L). In addition, they were characterized by high rates of resistance to netilmicin (*n* = 49, 75.4%), kanamycin (*n* = 54, 83.3%), gentamicin (*n* = 61, 93.9%), tetracycline (*n* = 60, 92.4%), ciprofloxacin (*n* = 55, 84.8%) and cotrimoxazole (*n* = 62, 95.5%), and a high rate of susceptibility to amikacin (*n* = 64, 98.5%). The double-disc synergy test was positive for all strains. Older patients were more susceptible to C3G-R Enterobacteriaceae infection than younger ones (*p* = 0.04) (Table 1).

**Table 1:** **Risk factors for infection with Enterobacteriaceae resistant to third-generation cephalosporins in 151 patients**

	**Presence (n = 65)**	**Absence (n = 86)**	**Univariate analysis**	
			**Crude OR**	***p***
Age (years)				
Mean	38.6	31.7		
CI	34.6–42.7	28.9–34.5	1.03	0.04
Gender, %				
Female	45.9	54.1	0.79	0.48
Preoperative antibiotic prophylaxis^a^, %				
Yes	46.3	53.7	1.27	0.48
Postoperative antibiotic prophylaxis^b^, %				
Yes	43.6	56.4	1.27	0.48
Surgery in past 12 months, %				
Yes	45.0	55.0	1.14	0.69
Hospitalization in past 12 months, %				
Yes	35.3	64.71	0.69	0.49
Altemeier wound class, %				
1	42.7	57.3		0.93
2	44.4	55.6		
3–4	42.8	57.2		
American society of anesthesiologists physical status score, %				
1	42.6	57.4		0.45
2	37.0	62.0		
3	66.7	33.3		
Length of operation, %				
<30min	39.0	60.0		0.25
30 min–1 h	41.3	58.7		
1–2 h	46.5	53.5		
>2 h	75.0	25.0		
Type of surgery, %				
Urology	50.0	50.0		0.31
Orthopaedics	51.8	48.2		
General surgery	34.6	65.4		
Gynaecology	40.0	60.0		
Delay between surgery and surgical-site infection (days), %				
<15	43.8	56.2		0.83
≥15	43.0	57.0		

OR, odds ratio.

^a^Antibiotics administered ≤ 60 min before the start of surgery.

^b^Antibiotics administered immediately after surgery.

### Antibiotic resistance determinants and replicons in C3G-R Enterobacteriaceae strains

The *bla*_CTX-M-15_ and *aac(6′)-Ib-cr* genes were detected in all strains. Of the 64 (98.5%) *qnr*-positive strains, 62 were *qnrB*, 56 were *qnrS* and none were *qnrA*, with 55 strains each harbouring 2 *qnr* genes.

### REP-PCR, ERIC-PCR and multilocus sequence typing of C3G-R Enterobacteriaceae

REP- and ERIC-PCR analysis showed 15 patterns for *E. coli* strains, 6 for *Enterobacter cloacae*, 5 for *K. pneumoniae* and 2 for *P. mirabilis*. Three clones were found in at least two surgical departments in the same hospital and six clones in at least two different tertiary care centres: two *Enterobacter cloacae* clones, three *K. pneumoniae* clones and three *E. coli* clones (Tables 2 and 3).

**Table 2:** **Molecular characteristics of third-generation cephalosporin-resistant** ***Escherichia coli*** **associated with surgical site infection**

**Sequence type (n)**	**ERIC- and REP-PCR based pattern (n)**	**Hospital/surgical department (n)**	**ESBL type (n)**	**PMQR determinants** ^**a**^ **(n)**
10 (12)	A (8)	A/GS (6), A/G (1), A/U (1)	CTX-M15 (8)	QnrB (8), QnrS (4), AAC(6′)-1b-cr (8)
	B (2)	A/GS (1), C/O (1)	CTX-M15 (2)	QnrB (2), QnrS (2), AAC(6′)-1b-cr (2)
	C (1)	CP/GS (1)	CTX-M15 (1)	QnrS (1), AAC(6′)-1b-cr (1)
	D (1)	C/O (1)	CTX-M15 (1)	QnrB (1), QnrS (1), AAC(6′)-1b-cr (1)
146 (1)	E (1)	A/GS (1)	CTX-M15 (1)	QnrB (1), QnrS (1), AAC(6′)-1b-cr (1)
131 (12)	F (1)	A/GS (1)	CTX-M15 (1)	QnrB (1), AAC(6′)-1b-cr (1)
	G (3)	A/GS (3)	CTX-M15 (3)	QnrB (3), QnrS (3), AAC(6′)-1b-cr (3)
	H (1)	C/O (1)	CTX-M15 (1)	QnrB (1), QnrS (1), AAC(6′)-1b-cr (1)
	I (1)	A/GS (1)	CTX-M15 (1)	QnrB (1), QnrS (1), AAC(6′)-1b-cr (1)
	J (5)	C/O (3), A/GS (1), CP/GS (1)	CTX-M15 (5)	QnrB (4), QnrS (4), AAC(6′)-1b-cr (5)
	K (1)	A/U (1)	CTX-M15 (1)	QnrB (1), QnrS (1), AAC(6′)-1b-cr (1)
156 (5)	L (5)	CP/GS (3), C/0 (2)	CTX-M15 (5)	QnrB (5), QnrS (5), AAC(6′)-1b-cr (5)
354 (1)	M (1)	CP/GS (1)	CTX-M15 (1)	QnrS (1), AAC(6′)-1b-cr (1)
405 (2)	N (1)	CP/GS (1)	CTX-M15 (1)	QnrB (1), QnrS (1), AAC(6′)-1b-cr (1)
	O (1)	A/GS (1)	CTX-M15 (1)	QnrB (1), QnrS (1), AAC(6′)-1b-cr (1)

A/GS, Amitié Hospital/general surgery; A/G, Amitié Hospital/gynaecology; A/U, Amitié Hospital/urology; C/O, Community Hospital/orthopaedics; CP/GS, Complexe Pédiatrique/general surgery; ESBL, extended-spectrum beta-lactamase; PMQR, plasmid-mediated quinolone resistance.

**Table 3:** **Molecular characteristics of 32 third-generation cephalosporin-resistant non-** ***Escherichia coli*** **Enterobacteriaceae associated with surgical-site infections**

**Species (n)**	**ERIC and REP-PCR-based pattern (n)**	**Hospital/surgical department (n)**	**ESBLtype (n)**	**PMQR determinants (n)** ^**a**^
*Enterobacter cloacae* (16)	A (1)	A/GS (1)	CTX-M15 (1)	QnrB (1), QnrS (1), AAC(6′)-1bcr (1)
	B (10)	A/G (5), A/GS (3), CP/GS (1), C/O (1)	CTX-M15 (10)	QnrB (10), QnrS (10), AAC(6′)-1bcr (10)
	C (1)	A/G (1)	CTX-M15 (1)	QnrB (1), QnrS (1), AAC(6′)-1bcr (1)
	D (1)	A/G (1)	CTX-M15 (1)	QnrB (1), QnrS (1), AAC(6′)-1bcr (1)
	E (2)	C/O (1), A/G (1)	CTX-M15 (2)	QnrB (2), QnrS (2), AAC(6′)-1bcr (2)
	F (1)	C/O (1)	CTX-M15 (1)	QnrB (1), QnrS (1), AAC(6′)-1bcr (1)
*Klebsiella pneumonia* (10)	A (3)	A/G (1), A/U (1), C/O (1)	CTX-M15 (3)	QnrB (3), AAC(6′)-1bcr (3)
	B (3)	C/O (3)	CTX-M15 (3)	QnrB, (3) QnrS (3), AAC(6′)-1bcr (3)
	C (2)	CP/GS (1), C/O (1)	CTX-M15 (2)	QnrB (2), QnrS (2), AAC(6′)-1bcr (2)
	D (1)	A/G (1)	CTX-M15 (1)	QnrS (1), AAC(6′)-1bcr (1)
	E (1)	A/G (1)	CTX-M15 (1)	QnrB (1), AAC(6′)-1bcr (1)
*Proteus mirabilis* (2)	A (1)	A/GS (1)	CTX-M15 (1)	QnrB (1), QnrS (1), AAC(6′)-1bcr (1)
	B (1)	C/O (1)	CTX-M15 (1)	QnrS (1), AAC(6′)-1bcr (1)
*Enterobacter amnigenus* (1)	A (1)	A/G (1)	CTX-M15 (1)	QnrB (1), QnrS (1), AAC(6′)-1bcr (1)
*Enterobacter sakazakii* (1)	A (1)	C/O (1)	CTX-M15 (1)	QnrB (1), QnrS (1), AAC(6′)-1bcr (1)
*Klebsiella oxytoca* (1)	A (1)	A/G (1)	CTX-M15 (1)	QnrB (1), QnrS (1), AAC(6′)-1bcr (1)
*Morganella morganii* (1)	A (1)	C/O (1)	CTX-M15 (1)	QnrB (1), QnrS (1), AAC(6′)-1bcr (1)

A/GS, Amitié Hospital/general surgery; A/G, Amitié Hospital/gynaecology; A/U, Amitié Hospital/urology; C/O, Community Hospital/orthopaedics; CP/GS, Complexe Pédiatrique/general surgery; ESBL, extended-spectrum beta-lactamase; PMQR, plasmid-mediated quinolone resistance.

^a^QepA was not found in any isolates.

Six multilocus STs were found in the 33 *E. coli* isolates: ST131 (six clones, *n* = 12), ST10 (four clones, *n* = 12), ST405 (two clones*, n* = 2), ST156 (one clone, *n* = 5), ST354 (one clone, *n* = 1) and ST146 (one clone, *n* = 1) (Table 2). All the ST131 isolates corresponded to the multi-resistant pandemic *E. coli* B2-O25b-ST131 group.

### Resistance transfer determination of C3G-R strains

Resistance transfer assays were performed on one strain randomly selected for each REP- and ERIC-PCR pattern. Transfer of *ESBL* by conjugation with *E. coli* J53-2 was successful for 22 (64.7%) of the 34 *ESBL* clones, which consisted of 17 *E. coli* isolates, 4 *Enterobacter cloacae* and 1 *Enterobacter amnigenus. ESBL* transfer by plasmid DNA electroporation into *E. coli* DH10B was successful for 5 (41.7%) of the 12 remaining isolates: 2 *K. pneumoniae* isolates, 2 *P. mirabilis* and 1 *Enterobacter cloacae*. The presence of *bla*_CTX-M-15_ and *aac(6′)-Ib-cr* was confirmed by PCR in the 27 transconjugants and transformants (Table 4). The plasmid harbouring the *bla*_CTX-M-15_ gene was systematically associated with *aac(6′)-Ib-cr*, in contrast to *qnr* genes, as no *qnrS* and 45.8% (11/24) of *qnrB* genes were co-transferred with *bla*_CTX-M-15_. Additional resistance genes (to aminoglycoside, chloramphenicol, nalidixic acid, tetracycline, sulfamide and trimethoprim-sulfamethoxazole) were probably transferred, as shown by antimicrobial susceptibility testing (Table 4).

**Table 4:** **Resistance genes transferred to transconjugants and electroporants and their replicon type**

**Species**	**ST**	**ERIC and REP-PCR based pattern** ^**a**^	**Hospital/department**	**PMQR determinants** ^**b**^	**Transconjugant or transformant** ^**c**^			
					**Incompatibility group**	**FII:FIA:FI formula**	**PMQR determinants** ^**b**^	**Non-beta-lactam drug resistance**
*Escherichia coli*	10	A	A/GS	QnrB, QnrS, AAC(6′)-1b-cr	IncF	F31:A4:B1	QnrB, AAC(6′)-1b-cr	GEN, TOB, NAL
*E. coli*	10	A	A/U	QnrB, AAC(6′)-1b-cr	IncF	F31:A4:B1	QnrB, AAC(6′)-1b-cr	GEN, TOB, NAL
*E. coli*	10	B	C/O	QnrB, QnrS, AAC(6′)-1b-cr	IncF	F31:A4:B1	AAC(6′)-1b-cr	KAN, GEN, TOB, CHL, TET, SUL, SXT
*E. coli*	10	C	P/GS	QnrS, AAC(6′)-1b-cr	IncF	F31:A4:B1	AAC(6′)-1b-cr	GEN, TOB, CHL, TET
*E. coli*	10	D	C/O	QnrB, QnrS, AAC(6′)-1b-cr	IncF	F31:A4:B1	AAC(6′)-1b-cr	KAN, GEN, TOB, CHL, TET, SUL, SXT
*E. coli*	146	E	A/GS	QnrB, QnrS, AAC(6′)-1b-cr	IncF	F36:A4:B1	AAC(6′)-1b-cr	GEN, TOB, CHL, TET
*E. coli*	131	F	A/GS	QnrB, AAC(6′)-1b-cr	IncF	F31:A4:B1	QnrB, AAC(6′)-1b-cr	KAN, GEN, TOB, CHL, NAL, TET, SUL, SXT
*E. coli*	131	G	A/GS	QnrB, QnrS, AAC(6′)-1b-cr	IncF	F31:A4:B1	QnrB, AAC(6′)-1b-cr	KAN, GEN, TOB, CHL, NAL, TET, SUL, SXT
*E. coli*	131	H	A/GS	QnrB, QnrS, AAC(6′)-1b-cr	IncF	F31:A4:B1	AAC(6′)-1b-cr	GEN, TOB, STR, TET, SUL, SXT
*E. coli*	131	I	A/GS	QnrB, QnrS, AAC(6′)-1b-cr	IncF	F1:A1:B20	AAC(6′)-1b-cr	GEN, TOB, CHL, TET, SXT
*E. coli*	131	J	A/GS	QnrB, QnrS, AAC(6′)-1b-cr	IncF	F36:A4:B1	AAC(6′)-1b-cr	KAN, GEN, TOB, NET, STR, CHL, TET, SUL, SXT
*E. coli*	131	J	C/O	AAC(6′)-1b-cr	IncF	F31:A4:B1	AAC(6′)-1b-cr	KAN, GEN, TOB, NET, STR, CHL, TET, SUL, SXT
*E. coli*	131	K	A/U	QnrB, QnrS, AAC(6′)-1b-cr	IncF	F31:A4:B1	QnrB, AAC(6′)-1b-cr	KAN, GEN, TOB, CHL, NAL, TET, SUL, SXT
*E. coli*	156	L	CP/GS	QnrB, QnrS, AAC(6′)-1b-cr	IncF	Fnew:A-:B20	AAC(6′)-1b-cr	KAN, GEN, TOB,SUL, SXT
*E. coli*	354	M	CP/GS	QnrS, AAC(6′)-1b-cr	IncF	F1:A1:B1	AAC(6′)-1b-cr	KAN, GEN, TOB, NET, STR, TET, SUL, SXT
*E. coli*	405	N	CP/GS	QnrB, QnrS, AAC(6′)-1b-cr	IncF	F31:A4:B1	AAC(6′)-1b-cr	GEN, TOB, CHL, TET
*E. coli*	405	O	A/GS	QnrB, QnrS, AAC(6′)-1b-cr	IncF	F36:A4:B1	QnrB, AAC(6′)-1b-cr	KAN, GEN, TOB, CHL, NAL, TET, SUL, SXT
*Enterobacter cloacae*	^d^	A	A/GS	QnrB, QnrS, AAC(6′)-1bcr	IncHI2	Not tested	QnrB, AAC(6′)-1bcr	KAN, STR, NAL, TET, SUL, SXT
*Enterobacter cloacae*	^d^	B	CP/GS	QnrB, QnrS, AAC(6′)-1bcr	IncF	Fnew:A-:B20	AAC(6′)-1bcr	KAN, TOB, STR, TET, SUL, SXT
*Enterobacter cloacae*	^d^	C	A/G	QnrB, QnrS, AAC(6′)-1bcr	IncF	F31:A4:B1	AAC(6′)-1bcr	GEN, TOB, STR, CHL, TET, SUL, SXT
*Enterobacter cloacae*	^d^	D	A/G	QnrB, QnrS, AAC(6′)-1bcr	IncF	F31:A4:B1	QnrB, AAC(6′)-1bcr	GEN, TOB, STR, CHL, NAL, TET, SUL, SXT
*Enterobacter cloacae*	^d^	E	C/O	QnrB, QnrS, AAC(6′)-1bcr	TF^e^			
*Enterobacter cloacae*	^d^	F	C/O	QnrB, QnrS, AAC(6′)-1bcr	IncF	F36:A4:B1	QnrB, AAC(6′)-1bcr	GEN, TOB, STR, CHL, NAL, TET, SUL, SXT
*Klebsiella pneumoniae*	^d^	A	A/U	QnrB, AAC(6′)-1bcr	TF			
*K. pneumoniae*	^d^	B	C/O	QnrB, AAC(6′)-1bcr	IncF	K4:- :B-	QnrB, AAC(6′)-1bcr	GEN, TOB, STR, NAL, TET, SUL, SXT
*K. pneumoniae*	^d^	C	CP/GS	QnrB, QnrS, AAC(6′)-1bcr	IncF	K4:- :B-	QnrB, AAC(6′)-1bcr	GEN, TOB, STR, NAL, TET, SUL, SXT
*K. pneumoniae*	^d^	D	A/G	QnrS, AAC(6′)-1bcr	TF			
*K. pneumoniae*	^d^	E	A/G	QnrB, AAC(6′)-1bcr	TF			
*Proteus mirabilis*	^d^	A	A/GS	QnrB, QnrS, AAC(6′)-1bcr	NT^f^			
*P. mirabilis*	^d^	B	C/O	QnrS, AAC(6′)-1bcr	NT			
*Enterobacter amnigenus*	^d^	A	A/G	QnrB, QnrS, AAC(6′)-1bcr	IncF	F31:A4:B1	AAC(6′)-1bcr	GEN, TOB, TET
*Enterobacter sakazakii*	^d^	A	C/O	QnrB, QnrS, AAC(6′)-1bcr	TF			
*K. oxytoca*	^d^	A	A/G	QnrB, QnrS, AAC(6′)-1bcr	TF			
*Morganella morganii*	^d^	A	C/O	QnrS, AAC(6′)-1bcr	TF			

TF, transfer to the *E. coli* recipient failed; NT, the plasmid could not be typed; ST, sequence type; PMQR, plasmid-mediated quinolone resistance, A/U, Amitié Hospital/urology; C/O, Community Hospital/orthopaedics; CP/GS, Complexe Pédiatrique/general surgery; A/GS, Amitié Hospital/general surgery; A/G, Amitié Hospital/gynaecology; KAN, kanamycin; GEN, gentamicin; TOB, tobramycin; NET, netilmicin; SRT, streptomycin; CHL, chloramphenicol; NA, nalidixic acid; TET, tetracycline; SUL, sulfamide; SXT, co-trimoxazole.

^a^Resistance transfer experiments were carried out on one randomly selected strain representative from all resistance gene content within each Rep- and ERIC-PCR banding pattern.

^b^QepA were not found in any of the isolates.

^c^ *bla*_CTX-M-15_ was found in all transconjugants and transformants.

^d^not tested.

^e^TF indicates that the transfer in the *E. coli* recipient failed.

^f^NT indicates that the plasmid was not typable.

### Genetic support of resistance determinants

PCR-based replicon typing in the 27 transconjugants and transformants demonstrated the presence of plasmids of the incompatibility groups IncF (*n* = 24, 88.9%) and IncHI2 (one *Enterobacter cloacae*) (Table 4). The plasmid in two *P. mirabilis* isolates could not be typed. In the incompatibility group IncF, identical replicon ST were found in most isolates: F31:A4:B1 (*n* = 14, 51.9%; 11 *E. coli*, 2 *Enterobacter cloacae* and 1 *Enterobacter amnigenus*) and F36:A4:B1 (*n* = 4, 14.8%; 3 *E. coli* and 1 *Enterobacter cloacae*). These two conjugative plasmids were found in strains collected from different hospitals. The other combinations were F1:A1:B1 (one *E. coli*), F1:A1:B20 (one *E. coli*), Fnew:A-:B20 (one *E. coli* and one *Enterobacter cloacae*) and K4:-:B- (two *K. pneumoniae*) (Table 4).

## Discussion

Enterobacteriaceae strains were the microorganisms most frequently associated with surgical-site infections, of which 65 (63.2%) were 3GC-R, as described previously in Africa [6]. Cross-transmission probably plays a major role, as suggested by the fact that patients in different surgery departments and different hospitals shared clonally related strains. This was the case not only for *K. pneumoniae* and *Enterobacter cloacae* strains, which are well known to be highly diffusible in hospital settings, especially in intensive care units [7], but also for *E. coli*, in contrast to the situation in developed countries [8]. Further investigations are needed to identify the mechanisms of transmission.

*E. coli* was the most frequent species among C3G-R Enterobacteriaceae. The 33 isolates belonged to six STs, of which three corresponded to at least two REP- and ERIC-PCR patterns, namely ST131, ST405 and ST10. The pandemic *E. coli* B2-O25b-ST131 group has contributed extensively to global dissemination of CTX-M-15 [2,9], as has ST405, with the dispersal of both CTX-M-15 and CTX-M-9 group enzymes [10]. In addition, ST10, which is typical in the human gut but is also responsible for intestinal and extra-intestinal infections, was recently associated with dissemination of CTX-M-1, CTX-M-2 and CTX-M-9 groups [10]. These data, combined with those from Nigeria, South Africa and Tunisia [11-13], show that dissemination of pandemic *E. coli* multi-drug resistant STs also occurs in African hospitals. The reasons for the wide dissemination and expansion of these clones are unknown but may include increased transmissibility, greater ability to colonize and/or persist in the intestinal tract, enhanced virulence and more extensive antimicrobial resistance than other *E. coli* [14]. The three minor STs have been described sporadically in humans and animals, associated with various CTX-M groups [15,16]: ST146 in Germany, ST156 in France, Great Britain and Portugal, and ST354 in Italy, Portugal and Spain. Five of the isolates found in two hospitals in this study were assigned to the ST156 genetic background, indicating its ability to spread.

Plasmid typing showed that horizontal gene transfer by IncF plasmid exchange was probably also involved in the dissemination of *bla*_CTX-M-15_. The ESBL *bla*_CTX-M-15_ gene was carried in all strains on plasmids of IncF incompatibility groups, except in one *Enterobacter cloacae*. IncF plasmids were the most prevalent in *E. coli* and *K. pneumoniae* carrying *ESBL* genes and also genes that confer resistance to tetracyclines, aminoglycosides and fluoroquinolones. These plasmids can be considered pandemic, as they have been detected in different countries and in bacteria of different origins and sources. The occurrence of these plasmid types appears to be closely linked to positive selection exerted by antibiotic use, incrementing their prevalence over that in bacterial populations that are not preselected for antimicrobial resistance. Their occurrence is also linked to virulence factors that contribute to the fitness of their bacterial host [17,18]. In the majority of isolates, including the three major *E. coli* STs, *bla*_CTX-M-15_ genes were found within IncF conjugative plasmids, with identical replicon ST formulae (F31:A4:B1 and F36:A4:B1), two plasmids reported to be abundant in humans [19]. The F31 and F36 alleles differ by only a single point mutation. This, in the context of the diversity of the REP- and ERIC-PCR patterns of the isolates and their presence in different species and in different hospitals, suggests that horizontal transfer of plasmids is probably an important mechanism of *bla*_CTX-M-15_ spread, as described previously [20]. The dissemination of plasmids was probably preceded by the centre-to-centre transmission of several strains by patient transfer, as indicated by the identification by REP- and ERIC-PCR of closely related *K. pneumoniae*, *E. coli* and *Enterobacter cloacae* isolates in at least two different hospitals.

Various sets of antibiotic resistance genes within F31:A4:B1 and F36:A4:B1 plasmids indicate their capacity for gene rearrangement and their evolution into new variants. *ESBL bla*_CTX-M-15_ and *aac(6′)-Ib-cr* genes were inconsistently associated with *qnrB* on these plasmids and also with other resistance genes, as shown by antimicrobial susceptibility testing of transconjugants. This suggests the presence of a multidrug resistance region, as described for IncFII plasmids pEK499 and pEK516 isolated in the United Kingdom from the *E. coli* ST131 group and for the pC15-1a plasmid disseminated in *E. coli* STs in Canada [20]. Further mapping of this region is needed. Frequent association of *bla*_CTX-M-15_, *qnrB* and *aac(6′)-Ib-cr* genes has been described in *K. pneumoniae* isolates in North, West, Central and East Africa, consistent with the hypothesis that these resistance-determinant genes are carried together on the same plasmid [21]. Such accumulation of resistance gene determinants on the same plasmid and their dissemination is a matter of concern, especially in countries with inadequate health care systems, because of the shortage of effective antibiotics and the consequences with respect to mortality, length of hospital stay and hospital costs. In addition, uncontrolled use of antimicrobial agents through self-medication, inappropriate antibiotic prescription and the substandard quality of some drugs favour the spread of antimicrobial resistance in these countries. The emergence of carbapenem resistance, which has been described in Africa [22] but was not found in our study, could be a serious challenge for infection control and antibiotic therapy in the future, as carbapenems are often the most effective antibiotics against 3GC-R Enterobacteriaceae.

## Conclusions

This study is of particular importance because of the difficulty of carrying out such studies in hospitals in countries with inadequate health care systems. Our data suggest that diverse modes of transmission of resistance are involved, probably with a major role of plasmid dissemination. All necessary measures should be taken in African hospitals to prevent nosocomial infections and the selection of resistant bacteria, with efficient nosocomial infection surveillance programmes. Standard hygiene and especially adherence to hand hygiene policies are the cornerstone for preventing transmission of multidrug-resistant bacteria.

## Methods

### Ethical clearance

The study protocols were approved by the National Ethics Committee of Central African Republic. Written informed consent to participate in the study was obtained from all patients.

### Patients

Patients with a surgical-site infection were recruited consecutively into the study between April 2011 and April 2012 in five surgical departments in three major tertiary care centres in Bangui: the Complexe Pédiatrique, the Amitié Hospital and the Community Hospital. A surgical-site infection was defined as an infection that occurred at or near a surgical incision within 30 days of the procedure or within 1 year if an implant was left in place [23]. Patients who underwent surgery were followed daily until discharge and surveillance was extended to 30 days post-operation, at a scheduled visit to the surgeon. The number of patients who did not return to the hospital and were lost to follow-up is unknown. A specific, standardized medical questionnaire was completed to collect demographic data, medical history over the previous 12 months, preoperative and postoperative antibiotic prophylaxis or treatment, type of surgery, Altemeier wound class, the American Society of Anesthesiologists’ physical status score, length of operation and delay between surgery and surgical-site infection (Table 1).

### Microbiological analysis, antimicrobial susceptibility testing and detection of ESBL

After superficial cleaning of wounds with physiological saline, a specimen was collected by rotating a sterile swab across the surface of the lesion, specifically targeting moist and necrotic areas. The specimens were placed in sterile tubes without transport medium at 4°C immediately after sampling and were processed within 2 h at the Pasteur Institute medical laboratory in Bangui. Sheep blood, chocolate and bromocresol purple agar plates were inoculated, incubated aerobically at 37°C and examined after 24 h and 48 h. Obligate anaerobes were not isolated. Bacterial isolates were identified by standard microbiological methods. Antibacterial drug susceptibility was determined by the disc diffusion method on Mueller–Hinton agar (Bio-Rad, Marnes La Coquette, France), according to the guidelines of the French Society of Microbiology (http://www.sfm-microbiologie.org). Production of ESBL in C3G-R Enterobacteriaceae was detected by the double-disc synergy test [24] and the MICs of ertapenem by the E-test method (AB Biodisk, Solna, Sweden). The ertapenem cut-off values were used for categorization. Susceptible strains were defined by MIC ≤ 0.5 mg/L and resistant strains by MIC >1 mg/L. All C3G-R Enterobacteriaceae were kept for further molecular analysis.

### DNA extraction and detection of beta-lactamase and plasmid-mediated quinolone resistance genes

Genomic DNA was extracted with the QIAmp™ kit (Qiagen, Courtaboeuf, France). Previously described polymerase chain reaction (PCR) methods were used to screen for plasmid-encoded *bla*_CTX-M_ and *bla*_SHV_ beta-lactamase genes, the *aac(6′)-Ib* gene and the quinolone resistance *qnr*A/B/S and *qepA* genes [24]. *bla*_CTX-M_ and *bla*_SHV_ were then characterized by direct DNA sequencing of the PCR products. All *aac(6′)-Ib* positive strains were further analysed by digestion with BtsCl (New England Biolabs, Ipswich, Massachusetts, USA) of the PCR product to identify *aac(6′)-Ib-cr* [24].

### Repetitive extragenic palindromic PCR, enterobacterial repetitive intergenic consensus sequence PCR and multilocus sequence typing

In order to assess the closeness of clonal relations between isolates at the local level, repetitive extragenic palindromic-PCR (REP-PCR) and enterobacterial repetitive intergenic consensus sequence-PCR (ERIC-PCR) were performed with REP-1R, REP-2 T and ERIC-2, respectively, as described previously [25]. Strains with an identical ERIC-PCR and REP-PCR banding pattern were defined as clonally related.

Multilocus sequence typing of *E. coli* strains was performed as recommended (http://mlst.ucc.ie/) on one strain randomly selected for each of the identical REP- and ERIC-PCR banding patterns. All isolates of ST131 were analysed by duplex PCR targeting the *pabB* and *trpA* genes to determine whether the isolate belonged to the O25b-ST131 group [2].

### Transferability of extended-spectrum beta-lactamase

Resistance transfer experiments were carried out on one randomly selected strain that was representative of the resistance gene content of each REP- and ERIC-PCR banding pattern. Conjugations were performed on solid media with *E. coli* K12 J5 resistant to sodium azide as the recipient strain [24]. Transconjugants were selected on Drigalski agar (Bio-Rad) supplemented with sodium azide (500 mg/L) and ceftazidime (4 mg/L). Transfer experiments by electroporation were performed for non-conjugative plasmids. Plasmid DNA from donors was extracted with a Qiagen plasmid midi kit (Qiagen, Courtaboeuf, France). Purified plasmids were used to transform *E. coli* DH10B (Invitrogen, Cergy-Pontoise, France) by electroporation according to the manufacturer’s instructions (Bio-Rad). Transformants were incubated at 37°C for 1.5 h and then selected on Drigalski agar (Bio-Rad) supplemented with 2.5 μg/mL cefotaxime. Transconjugants and transformants were tested for *ESBL* production followed by PCR amplification of the *ESBL* and plasmid-mediated quinolone resistance genes, as well as by plasmid replicon typing.

### Plasmid replicon type determination

PCR-based replicon-typing analysis was performed as described previously [26]. Plasmids belonging to IncF were typed according to a replicon sequence typing scheme [27].

### Data analysis

Microsoft Access 2003 was used for data entry, and Stata version 11 for statistical analysis. The chi-squared test and Student’s *t* test were used to compare categorical and continuous variables in univariate analysis, respectively. We considered *p* values < 0.05 to indicate significant associations.

## Abbreviations

ERIC-PCR: Enterobacterial repetitive intergenic consensus sequence-PCR
ESBL: Extended-spectrum beta-lactamase
3GC-R: Third-generation cephalosporin-resistant
Inc: Incompatibility group
MIC: Minimum inhibitory concentration
PCR: polymerase chain reaction
REP-PCR: Repetitive extragenic palindromic-PCR
ST: Sequence type
